# Experimental Evaluation of the Efficacy of Air-Sanitizing Equipment in Neutralizing Airborne SARS-CoV-2 Virus

**DOI:** 10.3390/pathogens14111096

**Published:** 2025-10-28

**Authors:** Alberto Izzotti, Nicolò Ruzzarin, Oriana Ferrante, Alessandra Pulliero

**Affiliations:** 1 Department of Experimental Medicine, University of Genoa, 16132 Genoa, Italy; 2 Department of Health Sciences, University of Genoa, 16132 Genoa, Italy; nicolo.ruzzarin@edu.unige.it (N.R.); oriana.ferrante@edu.unige.it (O.F.); alessandra.pulliero@unige.it (A.P.)

**Keywords:** COVID-19, air sanitization, UV, filtration, SARS-CoV-2 inactivation, airborne infective diseases

## Abstract

Air-sanitizing equipment is a collection of protective devices using filtration and/or UV irradiation to entrap aerosol and kill viruses, to prevent the spread of airborne infective diseases in indoor environments. The aim of the herein reported experimental study was to evaluate the possibility of attenuating the environmental spread of the SARS-CoV-2 virus by sanitizing indoor air. Aerosols were generated from human throat swab samples containing viable wild-type SARS-CoV-2. These samples were introduced into a controlled airflow channel and collected in buffered saline, with or without air sanitization. The viral presence was evaluated by antigenic test and qPCR. 34 different types of air- sanitizers were tested for their ability to neutralize viral aerosols. All devices neutralized viral infectivity as evaluated by a antigen test, qPCR, and cell infectivity, except for the unit without filtration and using LED-UV instead of bulbs, which was ineffective at 5 min but effective after 10 min of treatment. The obtained results provide evidence that 97% of the tested sanitizing devices are effective in breaking down the airborne viral load of wild human SARS-CoV-2 virus, even at a very high concentration, with a single passage of air. These results provide evidence that high-quality air-sanitizing devices may be used as a preventive tool to prevent the risk of airborne infections in indoor environments.

## 1. Introduction

The COVID-19 endemic is an ongoing complex problem that must be addressed by multiple preventive tools.

Vaccination is a measure certainly necessary, but not sufficient, to counteract the spread of the virus.

Control of virus spread can be achieved through the integrated use of prevention strategies that are both specific and unspecific.

The vaccine is the paradigmatic measure of specific active prophylaxis. The term ‘active’ indicates the activation of the recipient’s immune system, which is triggered to initiate the immune response process leading to the production of protective humoral immunoglobulins and immunological memory. Vaccination has some intrinsic characteristics that make it a fundamental but not solitary tool in the prevention of infectious diseases. The first-administration vaccine (primary response) needs 3–4 weeks [1] to evoke the production of protective antibodies; in this period, the recipient is susceptible to infection. Another element is that the antibody response is characterized by the production of humoral immunoglobulins. In fact, the vaccine, being administered parenterally through injection, elicits the formation of antibodies in the blood of the recipient and in other immunocompetent areas (e.g., lymph nodes), but not in the sites of airborne-virus entry that are the external nasopharyngeal mucosae. This means that the complete vaccination course against SARS-CoV-19 is very effective at reducing the risk of systemic complications in internal organs (pneumonia: 95% protection index), but less effective (65% protection index) at decreasing the possibility of infection.

However, vaccination is a specific preventive measure, and its effectiveness largely depends on the antigenic match between circulating strains and the vaccine composition, making them very effective against the antigenic variant for which they are designed, but less effective against newly emerging variants, known as ‘escape mutants’.

Therefore, for effective control of SARS-CoV-2 circulation and prevention of possible future epidemic waves, specific prophylaxis measures must be complemented by non-pharmaceutical and environmental prevention measures. In Preventive Medicine, this integrated approach is known as the ‘dogma of complementarities’, which states that preventive strategies achieve maximal efficacy when used synergistically, rather than in isolation. Accordingly, even vaccinated subjects should continue to adopt nonspecific preventive measures to ensure maximum prevention efficacy.

Unspecific prophylaxis includes individual and collective measures. Individual measures comprise the use of personal protective devices (masks, gloves, shoe covers, hair covers, glasses) and chemoprophylaxis. Personal protective equipment limits both the emission and the inhalation of the virus containing aerosols generated from the oropharyngeal mucosae of infected subjects during exhalation. Chemoprophylaxis refers to the preventive administration of antiviral or antiseptic agents, to reduce the risk of infection in exposed individuals. Collective measures include limiting the number of people in indoor environments and improving air quality through adequate ventilation, air filtration, and air-sanitizing systems or devices.

European legislation establishes that collective protection measures must take precedence over individual protection (EU Regulation 2016/425, Directive 89/391/EEC).

The containment measures of airborne diseases in controlled closed environments are unspecific; that is, they are effective not against a single pathogen or a specific variant thereof, but against many variants and against many pathogens.

Technology makes currently available devices capable of improving air quality in confined spaces, especially from the microbiological and virological point of view. These devices are identified as indoor air sanitizers.

To effectively combat the spread of airborne diseases, a primary focus is on ensuring the safety of indoor environments. This involves implementing innovative air purification methods and establishing robust indoor air cleaning systems. A variety of technologies are currently employed for air sanitization, although not necessarily referring to SARS-CoV-2 but also to other airborne viruses. The most prominent are non-thermal plasma (NTP), ultraviolet (UV) light, filters incorporating antimicrobial materials, electrical ionization, and photocatalytic oxidation (PCO) [2]. Non-thermal plasma (NTP), also known as cold plasma, is created through electrical discharges in a neutral gas, resulting in a mixture of ions, electrons, and radical species. Cold plasma can act upon purified SARS-CoV-2 RNA and modify the spike S1 protein, thereby inhibiting the virus’s ability to attach to host cells [3]. A byproduct of NTP generation is ozone, a highly reactive oxygen species and powerful oxidizing agent. Ozone has shown considerable efficacy in destroying a broad spectrum of microbes, including SARS-CoV-2 [4]. Ultraviolet (UV) light, specifically UV-C irradiation, has also been extensively studied and proven to inactivate SARS-CoV-2 [5]. To further enhance the antimicrobial capabilities of filters, the base materials (such as activated carbon granules or natural/synthetic fibers) can be enhanced with various compounds [6]. This not only improves their effectiveness but also helps minimize filter clogging and prevents the release of particles back into the environment. For instance, silver nanoparticles (AgNPs) are well-known potent biocides. They have demonstrated significant efficacy against bacteria like *Staphylococcus aureus*, *Enterococcus faecalis*, and *Escherichia coli*, as well as against extracellular SARS-CoV-2 [7,8]. Air sanitizers that combine filtration with other disinfection technologies are generally considered more effective at combating airborne infectious agents than those relying solely on filtration [9]. However, despite extensive research into the antimicrobial activity of individual disinfection technologies, there is still a lack of precise data on how efficient commercial indoor air sanitizers truly are [10].

The use of indoor sanitizers represents a great opportunity for Preventive Medicine.

Whenever indoor sanitization is not implemented, the alternative strategy to limit the airborne viral load often results in restricting access to enclosed public spaces. This situation dramatically occurred during the “lockdowns” enforced during the acute pandemic waves of COVID-19. Such closures represent an extreme emergency measure that could potentially be delayed or avoided through the effective use of indoor sanitization systems.

In practice, a valid alternative to the use of air sanitizers is to increase the dilution of airborne viral load [11].

This goal may be achieved by increasing the air volume available per person by increasing ventilation. However, this approach is not always easily applicable. Indeed, large, forced-ventilation infra-structures are very complex and expensive, and certainly not quickly achievable in emergency epidemic situations. Furthermore, in many areas the quality of the outdoor air is worse than that of the indoor air, especially in winter when airborne viral diseases spread. The use of indoor air sanitizers is a valid tool to solve this problem by improving the air quality from both a chemical–physical and microbiological point of view.

In fact, in addition to microbial contamination, one of the most common chemical–physical airborne pollutants is fine particles with a diameter less than 2.5 microns [12]. These small particles can overcome the defenses of our respiratory system and reach their depths, where they activate pathogenic mechanisms, triggering diseases such as asthma and chronic bronchitis. The International Agency for Research on Cancer of the World Health Organization classified fine particulate matter as a lung carcinogen for humans [13]. Some air sanitizers can significantly reduce the fine particulate matter concentration in indoor environments through the repeated passage of air through filters characterized by reduced pores and by very convoluted channels, therefore being able to trap the fine particulate matter [14].

This environmental situation poses a remarkable health risk when people at higher risk such as patients, older adults, or children are in indoor environments such as hospitals, care facilities, and schools [15].

The SARS-CoV-2 virus uses the oral–nasal aerosol emitted by sick subjects or those with long-incubation disease as a vector to move from one subject to another, and to spread into the environment. The spreading of this aerosol (Flügge droplet) is a necessary condition to sustain the contagion. Due to the large size of its composing drops, a droplet contains significant quantities of desquamated cells from the mucous membranes of the upper respiratory tract, especially of the pharynx, which are the main targets and infective source for airborne viruses [16]. This transmission mechanism highlights the importance of air sanitization as a preventive measure against SARS-CoV-2 infection. In fact, the airborne transmission of SARS-CoV-2 requires both the presence of infectious viral particles and ambient conditions that allow virus-laden aerosols to persist in the environment. Increasing ventilation and maintaining interpersonal distance can reduce the airborne viral load, but these measures, taken alone, may not be sufficient. Additional interventions, such as air sanitization, can further decrease the concentration of contaminated aerosols and, consequently, the number of airborne virions perpetuating the chain of infection.

The main limit to the use of air sanitizers is the lack of experimental protocols to evaluate their efficacy in decreasing viral infectivity.

The aim of the herein reported experimental study is to define a reproducible protocol to evaluate the possibility of stopping or attenuating the environmental spread of the SARS-CoV-2 virus by sanitizing the indoor air. For the first time, multiple tests (antigenic, molecular) and also a biological test using susceptible cells (challenge test) have been used together to evaluate the real efficacy of air-sanitizing equipment at 360 °C.

## 2. Materials and Methods

### 2.1. Sample Collection

A pool of 15 human throat-swab samples containing viable wild SARS-CoV-2 virus were collected for use to generate an aerosol. Informed consent was obtained from all subjects at the University San Martino Hospital, Genoa, Italy. Out of these 15 samples, 10 were positive for the Omicron SARS-CoV-2 variant and 5 for the Delta variant. The generated infected aerosol reproduced the airborne vector of the infecting virions as it would occur within an indoor environment containing 15 people. This aerosol was inserted into a channeled airflow and collected into fluid samples consisting of buffered saline mixtures that reproduced the pharyngeal fluid of the accepting subject. The ability of these samples to infect susceptible VERO cells with specific receptors for this virus was then verified. The presence or absence of the virus within these cells was evaluated by Polymerase Chain Reaction (qPCR) amplifying and quantifying viral RNA. The biological test used was the ‘challenge test’ as previously described in our published paper [17]. This test uses cells with high expression of the ACE2 receptor that specifically binds the spike protein of the SARS-CoV-2 virus (VERO C1008 E6 African green monkey kidney cells, code BSCL87, Experimental Zoo-prophylactic Institute of Lombardia and Emilia Romagna Region, Ministry of Health, Brescia, Italy). Accordingly, this test can verify both the presence of the virus and its infectivity and pathogenic capacity.

All the experiments were carried out in a BSL3 biosafety laboratory under controlled environmental conditions (temperature 22 °C, relative humidity 60%) by trained personnel equipped with biohazard protective devices and suitable for the COVID-19 biological risk.

### 2.2. Aerosol Formation

Samples containing high loads of wild SARS-CoV-2 virus were used to generate aero-diffusible aerosols conveyed into a channeled air flow. The Pro Farma RF7 device (Meafarma, 80035 Nola, LA, USA) was used to generate the aerosol. This aerosol is normally used to deliver drugs into the respiratory system of patients. According to the available certification (TUV Rheinland LGA Products GmbH, Nuremberg, Germany) and the acknowledged European Community reference guidelines (EN-13544-1), this equipment generates aerosol particles with a diameter within the 3.78–4.58-micron range whose fraction is at least 63% of the generated aerosol. The flow rate generated was 0.23 mL/min.

Eight milliliters of liquid sample was used to generate nebulized aerosol for a time equal to 1 h. The generated aerosol was conveyed through a flexible polypropylene tube directly into the bubbler sampler (positive reference sample) or after passage into the air-sanitizer equipment to be tested. The whole system was set up under a biosafety hood in closed mode to avoid accidental spreading of the virus into the environment. Due to the remarkable dimensions of some of the equipment to be tested, the exposure system was set up on a table outside the safety hood, ensuring that the whole air circulation system was properly sealed. The whole procedure was performed in a negatively pressurized room as available in the BSL3 lab.

### 2.3. Aerosol Generation

A bubbling sampler was used to collect the generated aerosol. This sample draws the air containing the aerosol with a defined constant flow and introduces it by adding turbulent flow into a glass vial containing the storage collection liquid. This situation reproduces the method of transmission of airborne infectious diseases as the acceptor also contains a liquid substrate, as is the case of the human pharynx, whose mucosa is covered by fluid. The suction flow used was 12.5 L/min. The bubbling sampler used was the Air Cube Com 2-TH (AMS Analitica, Pesaro, Italy).

For the generation of the positive reference samples, the aerosol generated was channeled through polypropylene pipes sealed at the junctions from the generation (aerosol) to the collection (bubbling sampler) devices, and thus the two devices were in direct connection.

### 2.4. Evaluation of Air Sanitization Efficacy in Decreasing SARS-CoV-2 Infectivity

The ability of the air-sanitizer equipment to neutralize the viral airborne load contained in the generated aerosol was then verified. For this purpose, the aerosol flow was channeled into the device’s suction openings. The emitted air was then collected in a bag placed to cover and to seal the edges of the air-sanitizer equipment surrounding the emission grids of the processed air. The bag was deflated when the air-sanitizer equipment was off (standby mode) and inflated when the air-sanitizer equipment was on (operation mode), thus allowing direct control of the correct emission of the air flow processed by the instrument.

A variety of air sanitization devices were tested including filter-based, UV-based and filter–UV-based sanitization equipment (Table 1).

### 2.5. Endpoints Monitored

For each one of the 36 experimental conditions (34 devices, plus positive and negative reference controls), 3 independent endpoints have been analyzed to evaluate SARS-CoV-2 viral loads in samples before and after air sanitization:

(1) Antigenic test (Hotgene Biotech, 102629 Beijing, China);
(2) Molecular detection of viral RNA by Polymerase Chain Reaction (qPCR);
(3) Presence of whole infective virions able to infect sensitive VERO cells (challenge test).

qPCR tests have been performed in quadruplicate analyses targeting two viral amplicons (Orf1, N).

A total of 864 analyses were performed (36 experimental conditions × 3 end points × 4 replicates × 2 amplicons).

**Figure 1:**
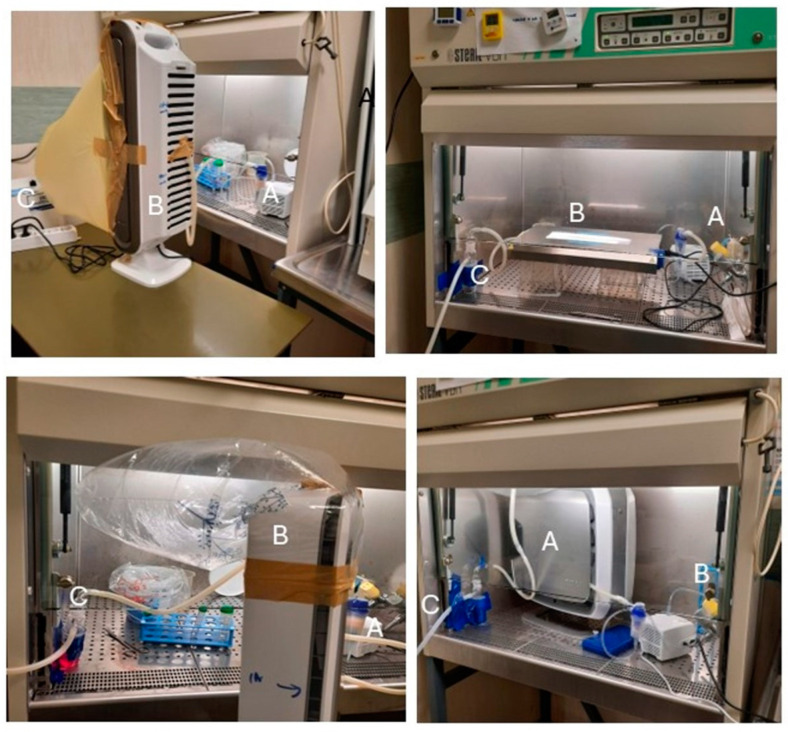
Experimental configurations of the equipment. Due to the large dimensions, some sanitization equipment cannot be located inside the safety hoods. Accordingly, that equipment was located on a table outside the hood using a sealed circuit to circulate inflow aerosol and outflow after air processing. A, aerosol generator; B, sanitizing equipment; C, bubbling collection sampler.

Examples of the operational configurations of the experimental system setup are shown in Figure 1.

### 2.6. Detection of SARS-CoV-2 RNA by qPCR Analysis

**Figure 2:**
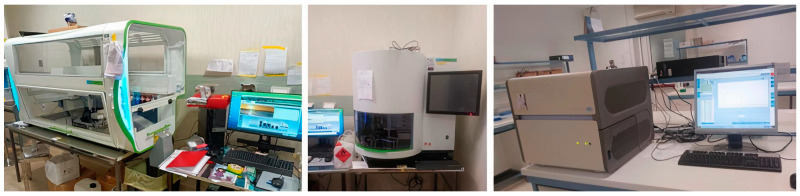
Equipment used for SARS-CoV-2 RNA analysis. RNA extraction was performed by high-performance robotic equipment using the Janus G3 (Perkin Elmer) preparatory robot (**left panel**) and magnetic bead extraction using the Chemagic 360 D (Perkin Elmer) automated equipment (**central panel**). RNA-DNA reverse-transcription and polymerization chain reaction (qPCR) was performed by using the Light Cycler II (Roche) high-sensitivity apparatus (**right panel**).

The presence of SARS-CoV-2 viral RNA was verified by RNA-DNA reverse-transcription (RT) and polymerization chain reaction (PCR) using the Light Cycler II (Roche, 6343 Rotkreuz, Switzerland) high-sensitivity apparatus. RNA extraction was performed by high-performance robotic equipment using the Janus G3 (Perkin Elmer, Shelton, CT, USA) preparatory robot and magnetic bead extraction using the Chemagic 360 D (Perkin Elmer) automated equipment (Figure 2).

For the analysis of each sample, fluorescent molecular probes (Roche) were used for the following genes: (a) house-keeping Ribonuclease P/MRP Subunit P30 [RPP30] used as a positive internal control to verify the presence of RNA and the correct implementation of the reaction qPCR; (b) viral SARS-CoV-2 Open Reading Frame (Orf) Orf1ab gene labeled with Vic fluorescent probe; (c) SARS-CoV-2 N viral gene labeled with FAM fluorescent probe. The following time/temperature conditions of qPCR amplifications were used: 50 °C × 15 min, 95 °C × 2 min, 45 cycles at 95 °C × 3 sec and 60 °C × 30 sec.

The experimental procedure used is the same as those currently used for the diagnosis of SARS-CoV-2 infection and respects all the qualitative, sensitivity, specificity and accuracy criteria.

The system was quantitatively calibrated making reference to a standard suspension of SARS-CoV-2 virus 1 × 10^7^ TCID50/mL producing qPCR positivity after 27 replication cycles.

### 2.7. Detection of SARS-CoV-2 Infectivity by Challenge Test

VERO cells expressing the high-affinity ACE2 receptor for the SARS-CoV-2 spike protein were incubated overnight at 37 °C with bubbling samples either without or with air sanitization according to the previously published challenge test protocol [14].

At the end of the incubation, the plates were heated at 57 °C for 30 min in a hybridization oven to inactivate the extracellular virus and detach the cells from the adhesion surface.

The cell suspension was then collected by scraping and aspiring with disposable Pasteur pipettes and centrifuged for 15 min at 3000× *g*. The collected cell pellet was re-suspended and washed 2 times with phosphate buffer (PBS) and re-centrifuged. The final collected pellet was then re-suspended in sterile RNAse-free molecular-grade double-distilled water and frozen until qPCR analyses.

**Figure 3:**
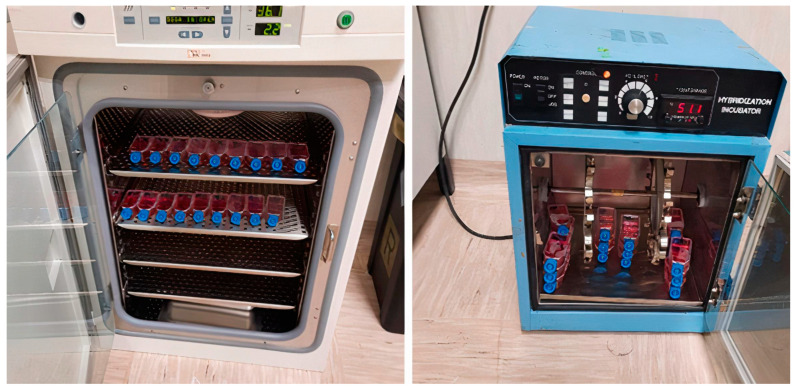
Experimental procedure used for testing sample infectivity by challenge test. Samples were incubated overnight with ACE2 expressing Vero cells (**left panel**) and virus-inactivated, and cells were detached by heating culture flasks in a hybridization oven; (**right panel**) cellular pellet collected by centrifugation and washing.

The procedure used for cell cultures is shown in Figure 3.

## 3. Results

An overall view of the results obtained is reported in Table 2. The table reports the results of the three monitored endpoints (columns) for each experimental condition tested (rows).

### 3.1. Antigenic Test

The antigen test indicated that SARS-CoV-2 was highly abundant in the positive reference sample, as demonstrated by the presence of both the viral antigen band (upper T line) and the control band (lower C line), with the T line darker than the C line.

The ELISA antigen test used is semi-quantitative and not quantitative. It is now reported that the high intensity of the positive result (semi-quantified as +++ in Table 2) corresponds to 1 × 10^7^ TCID50/mL. No other sample produced positive results in the antigen test.

The same sample after processing by air-sanitization equipment was fully negative. Indeed, only the control band appeared while the viral band was undetectable.

The full neutralization of the viral antigen was obtained by all air sanitization equipment tested, operating either by filtration, UV or their combination.

These findings indicate that the air sanitization process reduces or denatures SARS-CoV-2 antigens contained in the generated aerosol.

### 3.2. qPCR Analyses

**Figure 4:**
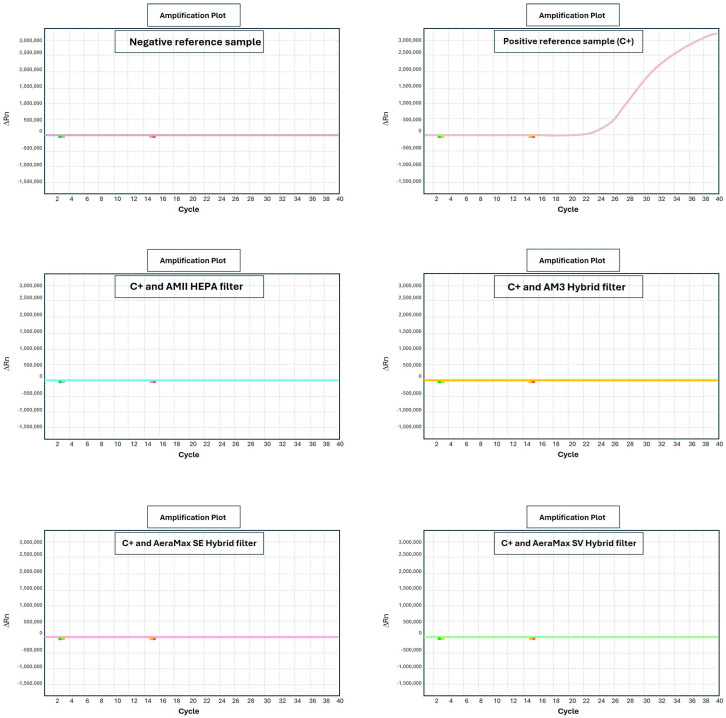
Examples of presence of viral nucleic acid before (C+) and after sanitization as evaluated by qPCR. Each panel corresponds to a specific experimental condition. Horizontal axis reports the number of qPCR amplification cycles. Vertical axis reports the intensity of fluorescence resulting from qPCR probe (ORF gene) binding the target.

Examples of results obtained by real-time PCR (qPCR) analyses for positive samples and for the same sample after sanitization are reported in Figure 4.

qPCR curves report the quantitative results obtained in terms of qPCR positivity cycle for each sample. Negativity threshold was established at 40 qPCR amplification cycles indicating that samples not giving any signal after this number of cycles do not have the presence of SARS-CoV-2 virus. The qPCR test is extremely sensitive, being able to detect only a single molecule of viral RNA. Accordingly, the negativity threshold of 40 qPCR cycles is very robust and stringent.

Samples collected from contaminated air (C+ aerosol) produced an intense fluorescent signal after 28 qPCR cycles.

The same sample, after processing by sanitizers equipped with either HEPA or Hybrid filter, underwent full negativization, with no signal being detectable even after 40 qPCR amplification cycles (>40th cycle, undetected).

From a quantitative standpoint, the difference in qPCR positivity cycle between untreated and treated samples was an average of at least 12 cycles. Because each cycle duplicates the viral RNA molecule, the obtained decrease in viral load was estimated to be 2^12^ corresponding to a 4096-fold reduction (>99%).

The only exception was the equipment using no filtration and UV produced by LEDs (instead of bulbs) when exposed for only short periods (5 min). Under this experimental condition a borderline positivity upon qPCR was detected at the 38th amplification cycle. Longer exposure times (10 min) resulted in viral neutralization under this experimental condition.

### 3.3. Challenge Test

**Figure 5:**
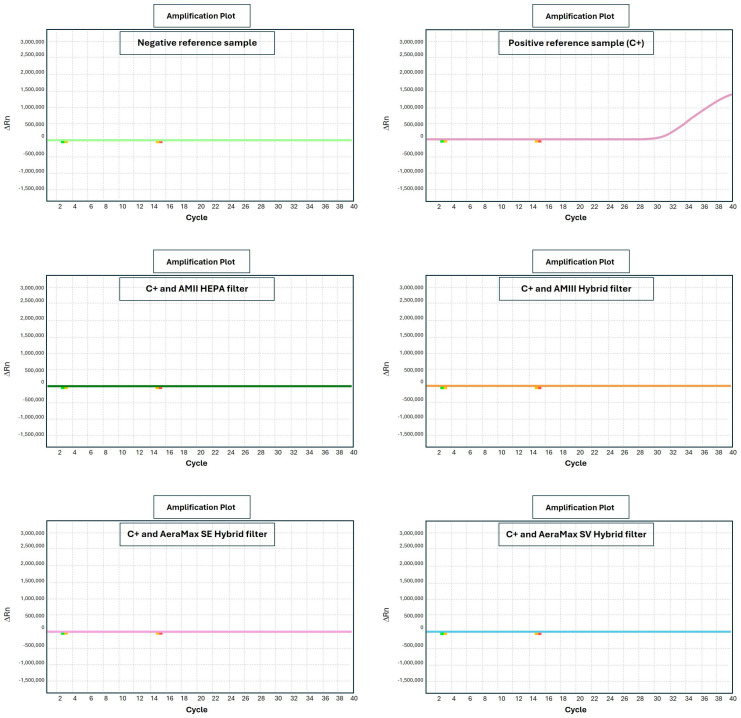
Infectivity before (C+, upper central panel) and after sanitization as evaluated by challenge test measuring the intracellular amount of viral nucleic acid by qPCR.

Results obtained from the challenge test and qPCR analyses for each equipment are reported in Figure 5.

The challenge test analyzes the ability of processed samples to transmit SARS-CoV-2 infection to sensitive cells.

The sample containing aerosolized wild-type live virus collected by a bubbling sampler constituted the positive reference sample without sanitization (aerosol C+). The intracellular viral load of SARS-CoV-2 in this positive sample was high with a positivity at cycle 31. By comparison, similar samples collected during the 2021–2022 epidemic produced positivity at the 30th amplification cycle.

After equipment processing, the same sample displayed fully negative results without the detection of any fluorescent signal, even after 40 qPCR amplification cycles (>40th cycle, undetected).

The only exception was the equipment using no filtration and UV as produced by LEDs (instead of bulbs) when exposed for only short periods (5 min). Under this experimental condition a borderline positivity upon qPCR was detected at the 36th amplification cycle. Longer exposure times (10 min) resulted in viral neutralization under this experimental condition.

### 3.4. Statistical Analyses

The variability range between replicate samples that underwent qPCR (reported as standard deviation) in Ct was <5%; i.e., replicate samples never produced differences >1 in detected Ct.

Quantitative comparisons were performed by Student’s t test for Ct data. The difference between the C+ sample and other samples (Ct 27 vs. 40) was significant (*p* < 0.01). In the challenge test, the difference between the C+ sample and other samples (Ct 31 vs. 40) was significant (*p* < 0.05). Quantitative comparisons were performed by Chi-square test for antigenic and challenge test results evaluating the positivity frequency in untreated (100%) vs. treated samples (4.3%) (*p* < 0.01).

## 4. Discussion

The experimentation carried out herein found that the sanitizing devices tested are effective in breaking down an airborne viral load of wild human SARS-CoV-2 virus, even at a very high concentration, with a single passage of air. It should be noted that the experimental conditions applied were extreme. In fact, we used a high viral load (qPCR positivity at cycle 20–30) [18]. This level of positivity is observed only in highly contagious patients. Furthermore, in field operational conditions, Flügge droplets emitted by the patient do not directly and completely impact the sanitizing devices but are considerably diluted in the ambient air before reaching the suction nozzle. Conversely, under our experimental conditions, the aerosol containing the high viral load was conveyed directly into the suction nozzle of the sanitation apparatus. These considerations lead to the conclusion that the tested devices have been evaluated under extreme laboratory operating conditions, certainly much more critical and difficult than those occurring in a real indoor environmental situation. Obviously, this situation was the desired one, since it is necessary that the effectiveness of similar sanitizing devices is tested in critical conditions much greater than real ones, to guarantee their effective performance.

The extremely challenging lab conditions, by far more stringent than those occurring in a real crowded indoor environment, are related to the following considerations:

(a) The whole generated aerosol directly impacted the equipment without any environmental dilution.
(b) The aerosol volume tested (8 mL) is by far higher than the amount of air droplets diffused in an indoor environment in 1 h.
(c) Only one sanitization passage into the equipment was performed while multiple sanitization passages occur in in-field conditions.
(d) Devices were set to their minimum capacity (minimum flowrate) to avoid excessive inflation of the collection bag and its possible rupture.
(e) The droplet diameter of the generated aerosol (3.78–4.58 µm range) was the most difficult to control. Under field conditions, droplets with larger and more heterogeneous diameters are produced.

Because of these reasons, the obtained lab results strongly support the evidence that the tested equipment is an effective tool for sanitizing air in in-field conditions. Indeed, despite the extreme conditions set, the tested sanitizing devices were able to reduce the airborne viral load as evaluated by all three endpoints analyzed (antigen test, qPCR, cell infectivity challenge test).

Nevertheless, our study has some limitations referring to (a) the limited availability of quantitative analysis of viral loads. Indeed, the antigenic and qPCR methods used are only semi-quantitative. Evaluation of viral loads analyzing the number of cell colonies infected could provide a better quantitative evaluation. Furthermore, electron microscope analysis in samples before and after sanitization could provide further experimental evidence.

These results are rather surprising for a filtering apparatus given the small size of the virion (<100 nm). However, it should be considered that the virion is contagious mainly when carried by Flügge particles, having a large diameter (droplets) [19]. The examined sanitizer, although not equipped with filters featuring pores with a diameter of less than 100 nm (which would make it impossible to sanitize adequate quantities of air in a reasonable time), effectively disrupts droplets and aerosol that facilitate SARS-CoV-2 transmission. Indeed, large droplets spreading airborne viruses are trapped in the filter inside the equipment. The herein-reported results demonstrate that the tested sanitizing devices are particularly effective in this regard. This situation is likely achieved thanks to the negative electric charge that is formed on the filters of high-quality sanitizers; this charge can trap the external components of the virus characterized by a strongly positive electric charge. SARS-CoV-2 is among the most gifted viruses in this sense. It is in fact the very high electrophilicity of its spike protein that makes it so contagious and able to rapidly bind to target cell receptors [20]. This situation allows quality sanitizers to greatly reduce the airborne viral load present in the air of a confined environment. However, despite the effectiveness of sanitization systems, SARS-CoV-2 can remain viable in aerosols for prolonged periods; therefore, airborne transmission cannot be completely ruled out. Several previously published studies have highlighted the persistence of SARS-CoV-2 in aerosols, with virions in aerosols able to survive for at least 3 h [21]. SARS-CoV-2 RNA has been detected in aerosol particles with diameters greater than 1 µm in hospital settings where COVID-19 patients were present [22]. Additionally, SARS-CoV-2 RNA has been found in aerosols at distances of at least 3 m from infected individuals in indoor environments, and in air pollution particles traveling through the air [23].

The greatest limit to the use of indoor sanitizers as a preventive practice is the lack of guidelines for accreditation and verification of their real effectiveness. Only sanitizers evaluated for their effectiveness under strictly controlled experimental conditions may be considered tools for the prevention of airborne diseases. COVID-19 in particular poses problems in this regard; in fact, it is not easy to have authorized laboratories able to handle this virus safely. However, this is necessary and possible although not particularly frequent. Today there are still very few sanitizers tested by accredited bodies for their ability to effectively break down the airborne viral loads of the SARS-CoV-2 virus.

The results of the many experiments performed have highlighted the quality of devices, making it possible to classify them as effective tools applicable to the prevention of airborne infectious diseases in confined environments, specifically including the prevention of COVID-19 disease.

Similar results have been previously obtained by other studies dealing with viruses and bacteria, including a study on the sanitization and decontamination of the surfaces in a bathroom using ultraviolet-C (UV-C) light technology [24]. The following microorganisms were analyzed: Methicillin-resistant Staphylococcus aureus (MRSA), Vancomycin-resistant Enterococcus faecium (VRE), Candida auris, Bacteriophage MS2 (a non-enveloped virus surrogate) and Clostridioides difficile spores. The results showed that MRSA, VRE, C. Auris and Bacteriophage MS2 infective load decreased by ≥1.2 log10 (range, 1.2 to 4.2 log10) at all test sites after 2 h of exposure to UV-C light. Aerosolized Bacteriophage MS2 was reduced by 4 log10 plaque-forming units in 45 min, confirming the good efficacy of the UV-C decontamination system. C. difficile spores were much more resistant, as their reduction was by <1 log10 after the same 2 h exposure.

Another previously published study [25] aimed to understand whether SARS-CoV-2 was present in public spaces, and to evaluate if cheap air purifiers with HEPA filters could be a useful tool for detecting the virus in the air. Samples were collected between February and May 2022 in six high-traffic areas (restrooms, waiting rooms, classroom) in community organizations in Miami. Thirty-two ProBreeze PB-P02 mini air purifiers (NW5 3EH London UK) with multi-layer filters were used for the collection of potential viral aerosols. Additionally, 35 swabs from high-contact surfaces were sampled. All samples deriving both from aerosols and swabs were analyzed in a laboratory using RT-qPCR to detect the presence of SARS-CoV-2. In total, 3 of the 32 air filters tested were positive for SARS-CoV-2 (all coming from a crowded place, i.e., a children’s day center) while none of the 35 surface swabs were positive, supporting the airborne transmission of the virus in indoor environments, and how HEPA filters are effective in both detecting the virus and reducing the risk of transmission, configuring them as a valuable tool in the monitoring of the transmission and the viral load.

A previously published study conducted on HCoV-229E, an alphacoronavirus known to cause disease in humans and widely used as a surrogate of SARS-CoV-2 in disinfection research, evaluated commercially available UV- and blue-light-based antimicrobial devices for their ability to kill the human coronavirus on surfaces and in air [26]. The results showed that two handheld UV devices brought about the complete inactivation of surface viral presence and a UV-C ceiling-based device caused a 1 log reduction in HCoV-229E in air, confirming the effectiveness of UV technology in sanitizing environments.

A study performed by Garg H. et al. investigated how UVC light inactivates aerosolized SARS-CoV-2 [27]. Two types of UVC-based air disinfection systems were designed and tested: one “in-duct” system for building or bus ventilation, and one “stand-alone” handheld unit for smaller spaces. In this study, live SARS-CoV-2 virus (Wuhan strain) was tested, although the collection of viruses using gelatine filters led to a loss of infectivity, due to the virus’s fragility. The virus was inoculated into highly receptive cells (Vero-E6-TMPRSS2) and its inactivation was measured using qPCR. The effects of UV-C light on virus spike proteins were also evaluated. Lastly, a Wells–Riley risk model has been applied to move the model from experimental to a real-world scenario, showing that the use of UV-C radiation could reduce the risk of infection in occupied spaces by up to 90%.

In another previously published study [28], the disinfection effect of a portable ionizer on SARS-CoV-2 (strain V34) and influenza A virus (strain CA04) was tested, and the results demonstrated that negative ions were significantly effective in reducing the concentration of particulate matter in the air above (over 63% efficiency) and in disinfecting the viruses stuck to the solid plate from the point of view of both nucleic acid and virus titer. The efficacy of decontamination was more than 99.8% after one hour of exposure and more than 87.77% after ten minutes of exposure. In addition, negative ions were effective also on aerosolized viruses, and they appeared to have a significant protective effect on susceptible animals exposed to viral aerosols, with an increased 50% infectious dose (ID50) over 4 times higher for animals exposed to viral aerosols (golden hamsters for SARS-CoV-2 and guinea pigs for influenza A virus). As regards safety, balb/c mice exposed to negative ions for 4 weeks had no abnormalities or lung pathologies, demonstrating that ions are a safe and effective tool to prevent airborne viral transmission.

Another study [29] evaluated the effectiveness of UV-C disinfection for SARS-CoV-2, considering the effects of the radiation on the environment and on the skin. A model to assess the right UV-C dose was designed, and UV-C at 254 nm and 222 nm and LED at 265 nm on SARS-CoV-2 (in PBS or EMEM + FBS) and a lentivirus (in DPBS, DMEM + FBS or artificial saliva) were tested. Disinfection of contaminated N95 masks and the UV-C absorption in fluids, amino acids and vitamins were also evaluated. Finally, the mechanical properties of the stratum corneum of human skin after UV-C exposure in different humidity conditions were examined.

The results showed how the suspension medium remarkably affects efficacy: SARS-CoV-2 in PBS had a susceptibility 4.4 times higher than in EMEM + FBS for UV-C at 254 nm, demonstrating an inverse correlation between the liquid absorbance and the viral susceptibility. Plus, the presence of amino acids and vitamins further reduced the susceptibility to the radiation, since they seem to absorb it. On N95 masks, the most effective length was 222 nm light. As regards the effects on the skin, no significant changes in elasticity were observed, but under high-moisture conditions, when the cumulative exposure at 222 nm exceeded 50 J/cm^2^, alterations in mechanical properties were described. These findings suggest maintaining the limit of the UV-C dose to the minimum needed for disinfection, with particular attention in public settings.

A study [30] conducted by Nardell E.A. was focused on germicidal ultraviolet light (GUV) to prevent airborne infection spread, especially for SARS-CoV-2.

The light length studied was 254 nm, due to its ability to damage RNA or DNA of pathogens.

In this study, the author underlines the importance of both natural and mechanical ventilation, also stating how those two are insufficient in healthcare facilities, since they cannot achieve the 6 to 12 room air changes per hour, which is recommended for airborne infection control. At the time of the study, there was still no evidence of SARS-CoV-2 being spread through ventilation systems and only two established room-based technologies were available to be added to mechanical ventilation, i.e., portable room cleaners and upper-room germicidal UV air disinfection.

Upper-room GUV appeared to provide the equivalent of 10 to 24 air changes per hour (ACH), way more efficient than standard mechanical ventilation. At the time of the study, lack of awareness about safety, efficacy and the airborne spread of SARS-CoV-2 was a limit to its application, stating the need to update guidelines.

In conclusion, the results presented by the herein reported experimental study and those made available from other performed studies demonstrate that the devices examined have remarkable performance in reducing the airborne contagious viral load of the wild human SARS-CoV-2 virus.

Therefore, these sanitizing devices represent a useful and effective safeguard as a non-specific prevention measure of contagion from airborne diseases in confined environments, with specific reference to the spread of the SARS-CoV-2 virus.

Many airborne viruses spread in the environment using air droplets like those processed in the herein presented study. Accordingly, it is likely that the same equipment is useful to attenuate the indoor environmental spread of many other airborne pathogens; thus, air sanitization represents a new important tool that is currently available for the unspecific prevention of infective disease.

## 5. Conclusions

Prevention of infective airborne disease is a complex task requiring a multi-layered approach including vaccines, protective masks, ventilation, distancing, hygiene, and surface cleaning. Air sanitization is an additional important tool in this context.

The biological implications of viral reduction percentages could also be included in the future in real-world indoor air safety standards (e.g., WHO or ASHRAE guidelines). However, quantitative thresholds are extremely difficult to be established for viruses compared with bacteria. Indeed, low viral loads can still be infective for susceptible and fragile subjects. Accordingly, in our opinion, the reference value should not be a quantitative threshold, instead considering negativity upon antigenic, qPCR and challenge tests.

In future, high-quality effective sanitizers should be distinguished from other low-quality equipment by performing these tests.

It is certain that in the next few years (or months) other pandemic waves will occur, such as occurring annually for *Orthomyxoviridae* (responsible for flu disease), or for the emergence of new ‘escape mutants’ (variants) of the SARS-CoV-2 virus. It is to be hoped that the experience and scientific knowledge accumulated in the last two years will allow us to finally face these situations not with passive restrictive measures such as lockdown, but with effective active prevention interventions in confined environments such as the installation of accredited air sanitizers.

## Figures and Tables

**Table 1:** The 34 air-sanitizer devices tested.

Code	Model	Manufacturer	Sanitization Method	Filter Type
1	Aeramax	Fellowes	Filtration	Standard
2	DX5	Fellowes	Filtration	Standard filter
3	DX55	Fellowes	Filtration	Standard filter
4	DX95	Fellowes	Filtration	Standard filter
5	ProAMII	Fellowes	Filtration	Standard filter
6	AMII	Fellowes	Filtration	HEPA filter
7	AM3	Fellowes	Filtration	Hybrid filter
8	AeraMax SE	Fellowes	Filtration	Hybrid filter
9	AeraMax SV1	Fellowes	Filtration	Hybrid filter
10	Array Stand AS1	Fellowes	Filtration	Standard Filter
11	Array Stand AS1	Fellowes	Filtration	Hybrid Filter
12	Array Stand AS2	Fellowes	Filtration	Standard Filter
13	Array Stand AS2	Fellowes	Filtration	Hybrid Filter
14	Array Wall AW1	Fellowes	Filtration	Standard Filter
15	Array Wall AW1	Fellowes	Filtration	Hybrid Filter
16	Array Wall AW2	Fellowes	Filtration	Standard Filter
17	Array Wall AW2	Fellowes	Filtration	Hybrid Filter
18	Array Ceiling AC2	Fellowes	Filtration	Standard Filter
19	Array Recess AR1	Fellowes	Filtration	Standard Filter
20	AeraMax SV2	Fellowes	Filtration	Hybrid filter
21	Z-1000	Accobrands	Filtration + UV	Standard filter
22	Z-2000	Accobrands	Filtration + UV	Standard filter
23	Z-2500	Accobrands	Filtration + UV	Standard filter
24	Z-3000	Accobrands	Filtration + UV	Standard filter
25	Z-3500	Accobrands	Filtration + UV	Standard filter
26	Z-6000	Accobrands	Filtration + UV	HEPA filter
27	Z-6000	Accobrands	Filtration + UV	Flu filter
28	Z-7000	Accobrands	Filtration + UV	HEPA filter
29	Z-7000	Accobrands	Filtration + UV	Flu filter
30	ASP-C2	Aureabeat	Filtration + UV	Standard filter
31	ASP-X1	Aureabeat	Filtration + UV	Standard filter
32	Air flux	Mectrotech	UV	No filter
33	Movie (5 min)	Mectrotech	UV led	No filter
34	Movie (10 min)	Mectrotech	UV led	No filter

**Table 2:** Results obtained by testing the aerosol samples pooled from 10 Omicron and 5 Delta SARS-CoV-2-positive oral swab samples either before (code 0) or after sanitization (codes 1–34). ‘+++’ corresponds to qPCR positivity at 28th Ct, ‘++’ at 36th Ct, and ‘+’ at 38th Ct.

Code	Sample	Antigenic Test	Molecular Test	Challenge Test (Infectivity)
0	Positive control	+++	+++	+++
1	Aeramax	—	—	—
2	DX5	—	—	—
3	DX55	—	—	—
4	DX95	—	—	—
5	ProAMII	—	—	—
6	AMII	—	—	—
7	AM3	—	—	—
8	AeraMax SE	—	—	—
9	AeraMax SV	—	—	—
10	Array Stand AS1	—	—	—
11	Array Stand AS1	—	—	—
12	Array Stand AS2	—	—	—
13	Array Stand AS2	—	—	—
14	Array Wall AW1	—	—	—
15	Array Wall AW1	—	—	—
16	Array Wall AW2	—	—	—
17	Array Wall AW2	—	—	—
18	Array Ceiling AC2	—	—	—
19	Array Recess AR1	—	—	—
20	AeraMax SV2	—	—	—
21	Z-1000	—	—	—
22	Z-2000	—	—	—
23	Z-2500	—	—	—
24	Z-3000	—	—	—
25	Z-3500	—	—	—
26	Z-6000	—	—	—
27	Z-6000	—	—	—
28	Z-7000	—	—	—
29	Z-7000	—	—	—
30	ASP-C2	—	—	—
31	ASP-X1	—	—	—
32	Air flux	—	—	—
33	Movie (5 min)	—	+	++
34	Movie (10 min)	—	—	—

—, negative result.

## Data Availability

The datasets used and/or analyzed during the present study are available from the corresponding author on reasonable request.
